# A Novel Identified Long Non-coding RNA, lncRNA MEF2C-AS1, Inhibits Cervical Cancer via Regulation of miR-592/RSPO1

**DOI:** 10.3389/fmolb.2021.687113

**Published:** 2021-06-08

**Authors:** Xiaoping Wang, Changhong Zhang, Meixuan Gong, Chen Jiang

**Affiliations:** Department of Gynaecology, Jinan Maternity and Child Care Hospital Affiliated to Shandong First Medical University/ Jinan Maternity and Child Care Hospital, Jinan, China

**Keywords:** cervical cancer, MEF2C antisense RNA 1, MiR-592, R-spondin 1, proliferation, migration, invasion

## Abstract

**Purpose:** Our purpose was to investigate the effect of lncRNA MEF2C antisense RNA 1 (MEF2C-AS1) on cervical cancer and further explore its underlying molecular mechanisms.

**Methods:** The proliferation, migration and invasion of CC cells were determined by counting Kit-8 (CCK-8), colony formation assay, and transwell assays, respectively. qRT-PCR and western blot were conducted to quantitatively detect the expression of lncRNA MEF2C-AS1, miR-592 and R-spondin1 (RSPO1). Kaplan-Meier survival curve from the Cancer Genome Atlas (TCGA) database and the Gene Expression Profiling Interactive Analysis (GEPIA) website was used to describe the overall survival. Bioinformatics analysis was performed to search the downstream target of lncRNA MEF2C-AS1 and miR-592. Luciferase reporter assay was conducted to detect the interaction between lncRNA MEF2C-AS1 and miR-592 or miR-592 and RSPO1.

**Results:** The data from GEPIA website showed that lncRNA MEF2C-AS1 expression was down-regulated in CC tissues and also associated with survival rate of CC patients. Moreover, the results of qRT-PCR also showed lncRNA MEF2C-AS1 was lowly expressed in CC cells. Subsequently, we confirmed that overexpression of lncRNA MEF2C-AS1 inhibited the proliferation, migration and invasion of CC cells. Further research illustrated that lncRNA MEF2C-AS1 was the target of miR-592, and RSPO1 was the downstream target gene of miR-592. Importantly, functional research findings indicated that lncRNA MEF2C-AS1 inhibited CC via suppressing miR-592 by targeting RSPO1.

**Conclusion:** In our study, we demonstrated the functional role of the lncRNA MEF2C-AS1-miR-592-RSPO1 axis in the progression of CC, which provides a latent target for CC treatment.

## Introduction

Cervical cancer is one of the most common gynecological malignancies second only to breast cancer, and is related to the high mortality and high incidence of women. The high incidence of carcinoma *in situ* was 30–35 years old, and the infiltrating carcinoma was 45–55 years old. In recent years, its incidence tends to be younger (Munksgaard and Blaakaer, 2011). As the most common subtype of cervical cancer, cervical squamous cell carcinoma (CESC) accounts for about 90% of cervical cancer cases (Ojesina et al., 2014; Bhatla et al., 2018). Despite great advances in surgery, chemotherapy and radiation therapy, and significant advances in treatment, there are still some early cases of invasion and metastasis, which directly affect the prognosis of CC (W. H. Organization, 2013). In addition, a considerable number of patients with CC are diagnosed as advanced stage and lack of effective treatment methods, leading to poor treatment effect (Kocaöz et al., 2018). Therefore, it is urgent to explore new potential markers for the treatment of CC and investigate the molecular pathogenesis of CC.

Long non-coding RNA (lncRNA) is an RNA transcript that is more than 200 nucleotides in length and does not contain protein coding capabilities (Gu et al., 2019). More and more studies have shown that the dysregulation of lncRNAs is a key determinant of tumor development (Zhang et al., 2014; Xie et al., 2019). As a matter of fact, lncRNAs have been regarded as a new gold mine for cancer treatment and diagnosis (Xie et al., 2019). In recent years, an accumulating number of researches have shown the key role of lncRNAs in the pathogenesis of CC. For example, Fan et al. (Fan et al., 2020) proved that LncRNA PTENP1 inhibited CC development by regulating miR-106b. Zhao et al. (Zhao et al., 2020) depicted that LncRNA GATA6-AS inhibited CC cells proliferation and promoted apoptosis by regulating miR-205. LncRNA MEF2C-AS1 is a novel identified long non-coding RNA. Previously, lncRNA MEF2C-AS1 was reported to be related with the proliferation and invasion of gastric cancer cells (Luo et al., 2018). However, whether lncRNA MEF2C-AS1 was involved in the progression of CC and what biological role it played had not been explored.

MicroRNAs (miRNAs), which are short, non-coding RNAs of approximate 20–23 nucleotides in length, regulate the expression of downstream genes at the post-transcriptional level (He et al., 2015; Wang and Cai, 2017). More and more data show that miRNAs occupy a key role in a variety of biological response, such as proliferation, apoptosis, invasion, migration and senescence (Gong et al., 2015; Abd El Gwad et al., 2018). For example, Gao et al. demonstrated that miR-592 inhibited the progression of glioma by regulating the expression of Rho-associated protein kinase (Gao et al., 2018). Besides, Fu et al. illustrated that miR-592, as an oncogene, promoted the development of human colorectal cancer tumors by targeting the expression of FoxO3A (Fu et al., 2016). However, the biological function of miR-592 in CC was rarely explored, let alone the underlying molecular mechanisms.

Herein, the aim of this study was to investigate the involvement of lncRNAs and miRNAs in CC. It was assumed that lncRNA MEF2C-AS1, inhibits CC via regulation of miR-592/RSPO1 axis.

## Materials and Methods

### Cell culture

Cervical cancer cells (Caski, C-33A, SiHa and HeLa) and normal cervical cell (Ect1/E6E7) were obtained from American Type Culture Collection (ATCC, Manassas, VA). After resuscitation, they were cultured in RPMI-1640 medium containing 10% fetal bovine serum (FBS, Beyotime, Beijing, China) in a humidified environment. When cells attained 70–80% confluences, transfection experiments could be conducted.

### Cell Transfection

The pc-MEF2C-AS1, pc-NC, miR-592 mimic, mimic NC, miR-592 inhibitor, inhibitor NC, si-RSPO1 and si-NC were constructed by GenePharm (Shanghai, China). SiHa and HeLa cells were seeded into a six-well plate at a density of 2 × 10^5^ cells/well. When the cell confluence reached 80%, 4 μg pc-MEF2C-AS1 (pc-NC) or 50 nM miR-592 mimics (mimics NC) was transfected into cells per well in the 6-well plate using Lipofectamine 3,000 (Invitrogen, United States). Then, per well in the 6-well plate, the cells were co-transfected with 4 μg pc-MEF2C-AS1 (pc-NC) and 50 nM miR-592 mimics (mimics NC) using Lipofectamine 3,000 (Invitrogen, United States). Moreover, 50 nM miR-592 inhibitor (inhibitor NC) and 50 nM si-RSPO1 (si-NC) were co-transfected into SiHa and HeLa cells using Lipofectamine 3,000 (Invitrogen, United States). After 48 h post-transfection, the transfection efficiency was measured by qRT-PCR.

### Quantitative Real-Time PCR

TRIpure reagent (Invitrogen, United States) was used to isolate the total RNA from samples and PrimeScript RT kit (TaKaRa, Otsu, Japan) was used for reverse transcription. After the sample was prepared, the expression level was detected with SYBR green, and GAPDH was controlled as internal parameter. 2^−ΔΔCt^ methods represented the fold changes of gene expression and the experiments were conducted three times. All primers were designed by GenePharma (Shanghai, China) and were as follows: lncRNA MEF2C-AS1 (sense): 5′-ACT​TGT​TGC​CTA​CTA​TCA​TAC​CTG-3′ (anti-sense): 5′-ATA​GCC​ATA​CAA​TAA​GTT​GCT​CT-3′; miR-592 (sense): 5′-TTG​TGT​CAA​TAT​GCG​ATG​ATG​T-3′ (anti-sense): 5′-GCG​AGC​ACA​GAA​TTA​ATA​GCA​C-3′; RSPO1 (sense): 5′-AAG​GCT​ACT​CTG​CTG​CCA​AC-3′ (anti-sense): 5′-CGA​TTT​CTG​TTC​CCG​TTT​GT-3′; U6 (sense): 5′-GCT​TCG​GCA​GCA​CAT​ATA​CTA​AAA​T-3′ (anti-sense): 5′-CGA​TTC​ACG​AAT​TTG​CGT​GTC​AT-3′; GAPDH (sense): 5′-ACA​GTC​AGC​CGC​ATC​TTC​T-3′ (anti-sense): 5′-GAC​AAG​CTT​CCC​GTT​CTC​AG-3′.

### Cell Counting Kit-8

The number of viable cells of SiHa and HeLa were detected by the CCK-8 (Beyotime, Beijing, China). In brief, the cells were inoculated on 96-well plates at a density of 1.0 × 10^4^ cells/well and treated accordingly. Then, each well was added with CCK-8 solution and incubated for 2 h in the dark.

### Colony Formation Assay

The same densities of SiHa and HeLa cells of each group were collected and cultured in 6-well plates. The medium was changed every 2–3 days and cultured continuously for 14 days. After washing the cells twice with PBS, the cells were fixed with 4% formaldehyde for 15 min at 37°C. Subsequently, residual formaldehyde was removed, and cell clones were stained with crystal violet for 10–20 min. Cells in each hole were observed under the microscope and the number of clones was counted.

### Transwell Assay

For the transwell migration assay, the SiHa and HeLa cells resuspended in serum-free medium were placed in the upper chamber of each insert (Corning, United States) containing the noncoated membrane. For the invasion assay, the transwell membrances were pre-coated with Matrigel (BD Biosciences, United States). Subsequently, the lower chamber was replaced by a medium supplement 15% fetal bovine serum (FBS, 600 μL). After incubation at 37°C for 24 h, 4% paraformaldehyde was used for fixation for 10 min and 0.1% crystal violet for staining for 30 min, respectively. The cells from five representative fields were counted under a microscope.

### Western Blot Analysis

According to the manufacture’s instruction, the proteins were extracted and its concentration was measured. Subsequently, the prepared protein was separated by polyacrylamide-SDS gels and then transferred onto PVDF membranes (Roche, Switzerland). After blocking, the PVDF membrane was subjected to incubation with primary antibodies: RSPO1 (1:1,000, Proteintech Group Inc., Wuhan, China). On the following day, the membrane were incubated with the secondary antibody at 37°C for 45 min and the intensity of protein expression was detected by ECL chemiluminescence (Beyotime, Beijing, China).

### Luciferase Reporter Assay

Dual-luciferase reporter vectors MEF2C-AS1-WT (wild type) and RSPO1-WT reporters were formed via cloning MEF2C-AS1 sequence or 3ʹ-UTR (untranslated region) of RSPO1, which both contained the miR-592 binding sites, into pmirGLO reporter vectors (Promega, Madison, WI, United States). The mutant vectors MEF2C-AS1-MUT (mutant) and RSPO1-MUT were designed using point mutations of miR-592 binding sites. SiHa and HeLa cells were plated onto 24-well plates and co-transfected with vectors the above constructed and miR-592 mimics or mimics NC. Luciferase activities were determined with the Dual-Luciferase Reporter System (Promega, Madison, WI, United States).

### Bioinformatics

The differential expressions of lncRNAs, miRNAs and mRNAs in CC tissues and their relative prognosis curve were analyzed in the Cancer Genome Atlas (TCGA) database (https://cancergenome.nih.gov/) and the Gene Expression Profiling Interactive Analysis (GEPIA) website (http://gepia2.cancer-pku.cn). Differential expressed genes (DEGs) was analyzed by using the Limma package or DESeq package. The DEGs were screened by the following criteria: |the log-fold change (log_2_FC)| > 1 and *p*-value < 0.05. Moreover, potential miRNAs that may bind to lncRNA MEF2C-AS1 was predicted by searching the DIANA tools (http://diana.imis.athena-innovation.gr/DianaTools/index. php?r = microT_CDS/index) and the differentially expressed miRNAs screened from TCGA database. Subsequently, the target genes of miR-592 were predicted by retrieving the TargetScan (http://www.targetscan.org/vert_71/), the differentially expressed mRNA screened from TCGA database and the mRNA with significant survival differences screened from TCGA database. Finally, the genes were intersected using Venn analysis (http://bioinformatics.psb.ugent.be/webtools/Venn/).

### Statistical Analysis

All the data were analyzed by Statistical Package for Social Sciences19.0 (SPSS, Chicago, IL, United States). One-way ANOVA followed by Dunnett’s multiple comparison was applied to assess the differences between the groups. *p* < 0.05 indicated significant difference between groups.

## Results

### LncRNA MEF2C-AS1 Was Low Expressed in CC

**FIGURE 1:**
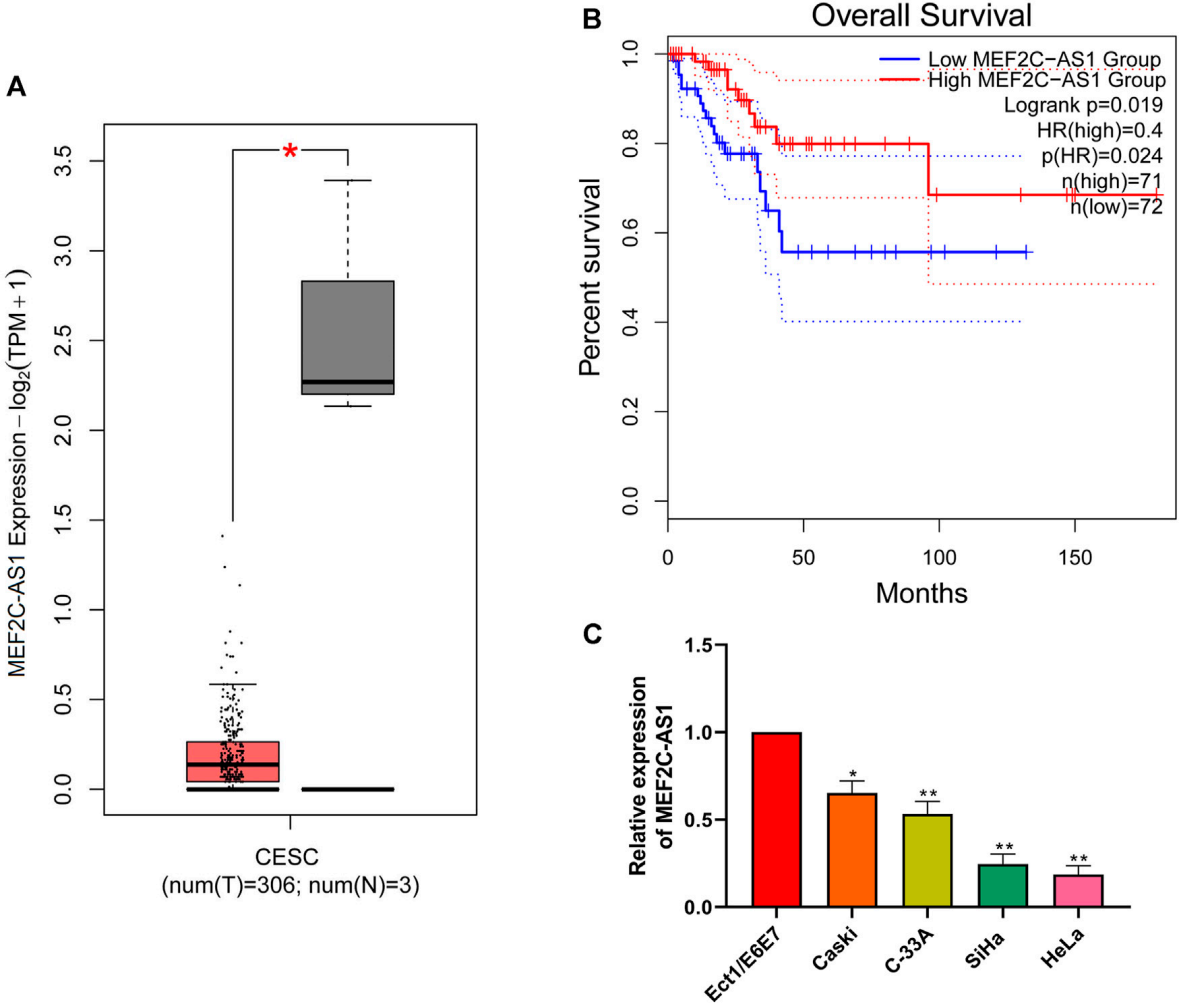
LncRNA MEF2C-AS1 was low expressed in CC. **(A)** The expression of lncRNA MEF2C-AS1 in CC tissues compared with the normal tissues in the GEPIA website; **(B)** The prognosis of CC individuals provided the GEPIA dataset (the criteria of two groups was Quartile); **(C)** qRT-PCR was performed to detect the lncRNA MEF2C-AS1 expression in cervical cancer cell lines (Caski, C-33A, SiHa, and HeLa) and normal cervical cell (Ect1/E6E7). Measurement data were expressed as mean ± standard deviation. The cell experiment is repeated three times independently. *p < 0.05 compared with normal tissues **(A)**; *p < 0.05, **p < 0.01, compared with Ect1/E6E7 group **(C)**.

The differential expression of lncRNA MEF2C-AS1 in CC was detected via GEPIA website. As indicated in Figure 1A, the expression of lncRNA MEF2C-AS1 in CC tissues was significantly lower than that in normal tissues (*p* < 0.05). Meanwhile, the data from GEPIA website showed that lower lncRNA MEF2C-AS1 expression was also associated with lower survival rate (*p* < 0.05) (Figure 1B). Further, we compared the transcriptional expression of lncRNA MEF2C-AS1 in CC cell lines (Caski, C-33A, SiHa, and HeLa) and normal cervical cell (Ect1/E6E7). As expected, the transcription level of lncRNA MEF2C-AS1 was evidently decreased in Caski (*p* < 0.05), C-33A (*p* < 0.01), SiHa (*p* < 0.01) and HeLa cells (*p* < 0.01), as compared with Ect1/E6E7 (Figure 1C). Besides, lncRNA MEF2C-AS1 expression in SiHa and HeLa cells was lower than Caski and C-33A cells, thereafter selected as conducting the following experiments.

### Overexpression of lncRNA MEF2C-AS1 Inhibited CC Cell Proliferation, Migration and Invasion

**FIGURE 2:**
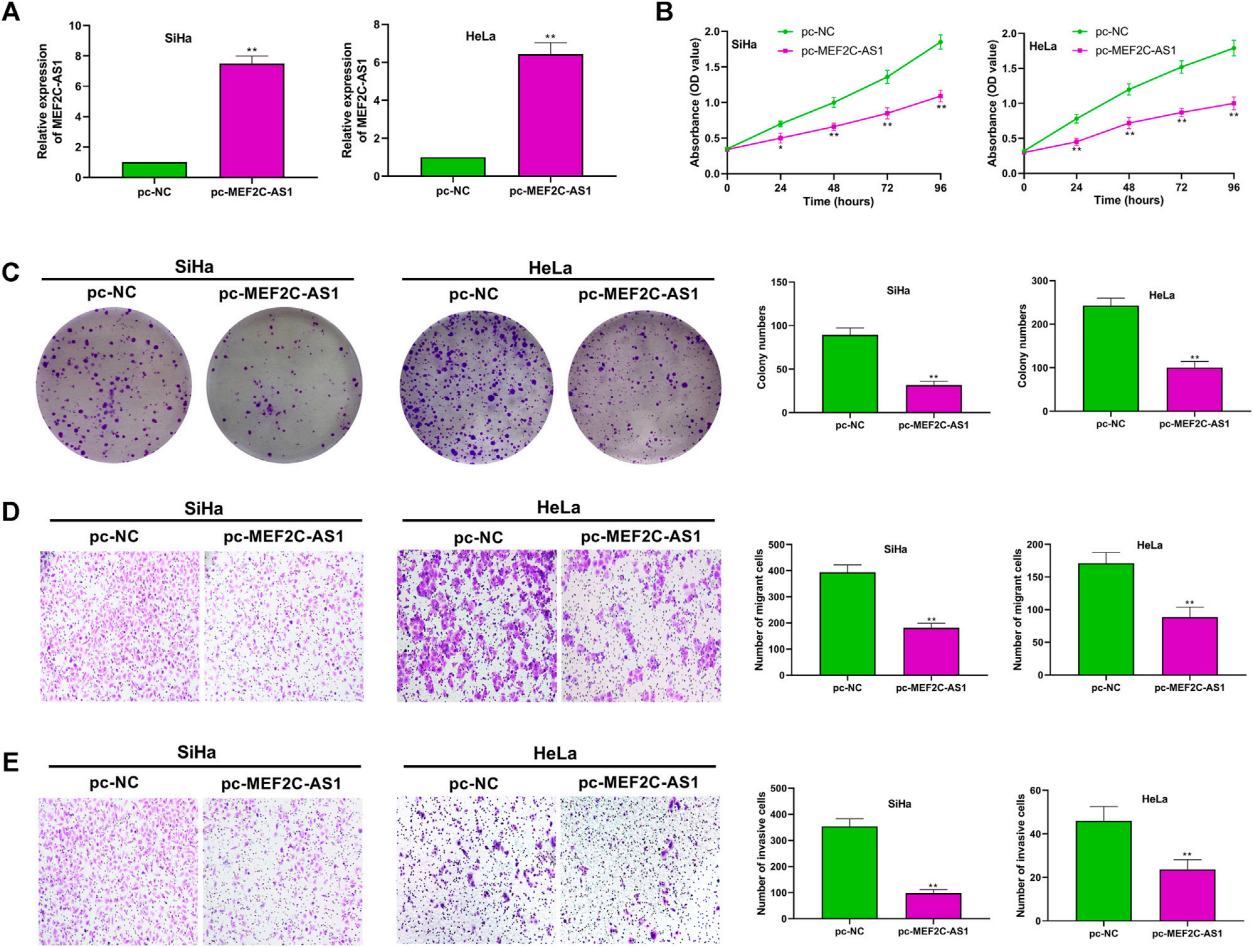
Overexpression of lncRNA MEF2C-AS1 inhibited CC cell proliferation, migration and invasion. **(A)** The expression efficiency of lncRNA MEF2C-AS1 was detected by qRT-PCR; **(B)** After transfection with pc-MEF2C-AS1, the proliferation ability of the SiHa and HeLa cells were measured by CCK-8; **(C)** After transfection with pc-MEF2C-AS1, the colony formation ability of SiHa and HeLa was measured by colony formation assay; **(D)** Transwell assay was conducted to detect the migration ability of SiHa and HeLa; **(E)** The invasion ability of SiHa and HeLa was measured by Transwell assay. Measurement data were expressed as mean ± standard deviation. The cell experiment is repeated three times independently. *p < 0.05, **p < 0.01, compared with pc-NC group **(A–E)**.

To investigate the role of lncRNA MEF2C-AS1 in CC, we overexpressed lncRNA MEF2C-AS1 and detected its overexpression efficiency (*p* < 0.01) (Figure 2A). Then, CCK-8 and colony formation assay were conducted to detect the effect of pc-MEF2C-AS1 on SiHa and HeLa proliferation, and the results showed that overexpression of lncRNA MEF2C-AS1 greatly inhibited cell growth (*p* < 0.05, *p* < 0.01) and clone formation (*p* < 0.01) (Figures 2B,C). Furthermore, we examined the effect of pc-MEF2C-AS1 on the migration and invasion of SiHa and HeLa cells. As shown in the transwell assay, the cell migration rate of pc-MEF2C-AS1 group was markedly lower than that of si-NC group (*p* < 0.01) (Figure 2D). Similarly, the invasion efficiency of cervical cancer cells was significantly inhibited after transfection with pc-MEF2C-AS1 (*p* < 0.01) (Figure 2E).

### LncRNA MEF2C-AS1 Was the Target of miR-592

**FIGURE 3:**
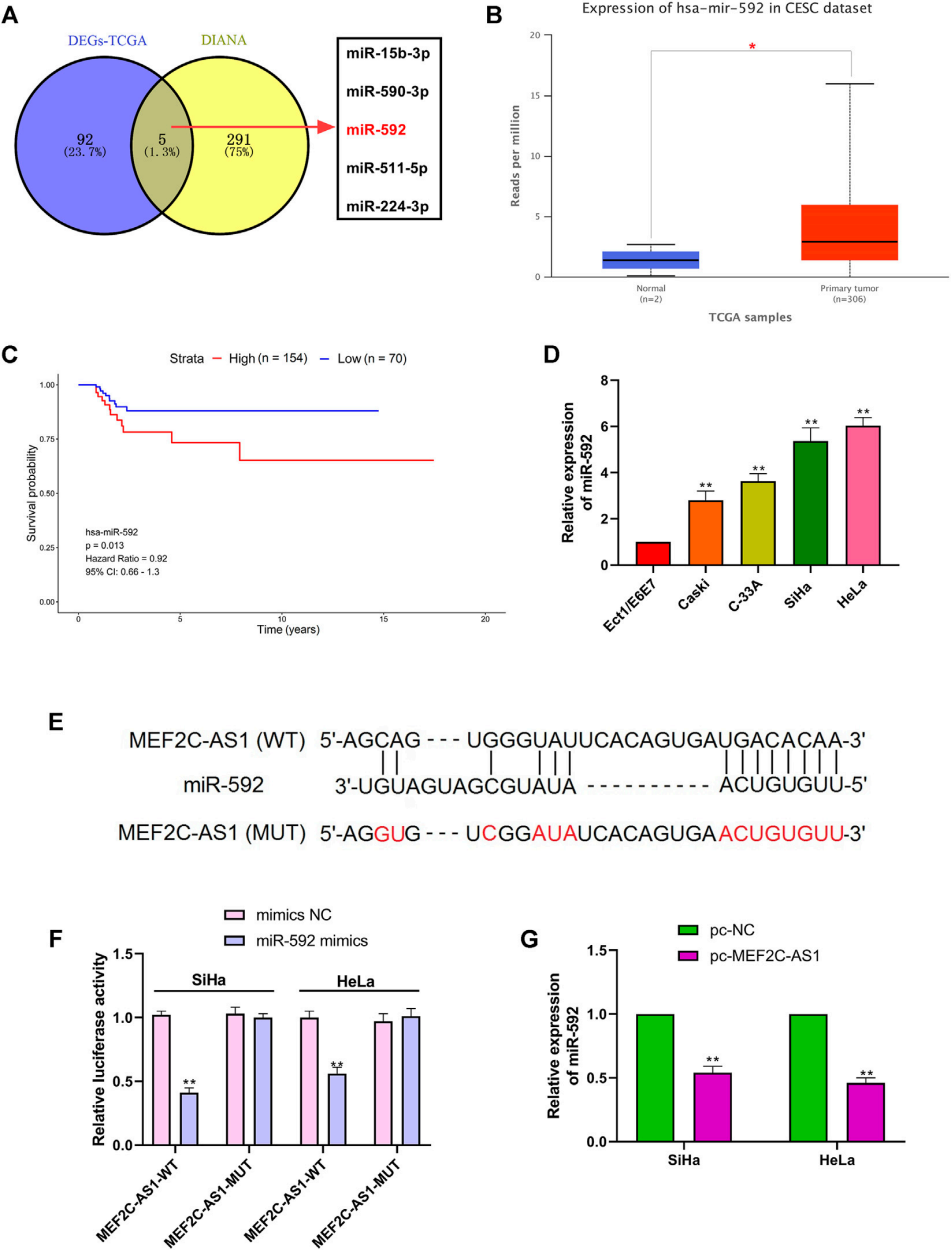
LncRNA MEF2C-AS1 was the target of miR-592. **(A)** The potential miRNAs that may bind to lncRNA MEF2C-AS1 was predicted by searching the DIANA tools and the differentially expressed miRNAs screened from TCGA database; **(B)** TCGA data showed the expression changes of miR-592 in CESC tissues; **(C)** Kaplan-Meier survival curve from TCGA described the overall survival; **(D)** qRT-PCR was performed to detect the miR-592 expression in cervical cancer cell lines (Caski, C-33A, SiHa, and HeLa) and normal cervical cell (Ect1/E6E7); **(E)** DIANA tool predicts the binding sites between lncRNA MEF2C-AS1 and miR-592; **(F)** luciferase activity of MEF2C-AS1-wt, MEF2C-AS1-mut upon the transfection of miR-592 mimics or mimics NC was evaluated by luciferase reporter assay; **(G)** The mRNA expression of miR-592 was detected in SiHa and HeLa cells by qRT-PCR after transfection with pc-MEF2C-AS1. *p < 0.05, compared with normal tissues **(B)**; **p < 0.01, compared with Ect1/E6E7 group **(D)**; **p < 0.01, compared with mimics NC group **(F)**; **p < 0.01, compared with pc-NC group **(G)**.

To elucidate the molecular mechanism of lncRNA MEF2C-AS1 in regulating the progression of CC, we conducted bioinformatics analysis. As presented in Figure 3A, the potential miRNAs that may bind to lncRNA MEF2C-AS1 was predicted by searching the DIANA tools and the differentially expressed miRNAs (DEmiRNAs) screened from TCGA database. Then, the Venn diagram of intersected candidate target genes was plotted, and the five DEmiRNAs were miR-15b-3p, miR-590-3p, miR-592, miR-511-5p, and miR-224-3p. As miR-592 plays a key role in many types of cancer (Lv et al., 2015; He et al., 2018; Pan et al., 2021), it was selected for further analysis. In the TCCA database, we found that the expression of miR-592 was sharply up-regulated compared with normal tissues (*p* < 0.05), but its high expression was negatively correlated with survival (Figures 3B,C). Further, we detected the expression level of miR-592 in CC cell lines and found that miR-592 was up-regulated in above cells (*p* < 0.01) (Figure 3D). Therefore, we speculate that lncRNA MEF2C-AS1may be a potential target of miR-592 and the binding sites were shown in Figure 3E. To confirm this conjecture, two types luciferase reporter gene vectors (MEF2C-AS1-wt and MEF2C-AS1-mut) were conducted. As depicted in Figure 3F, the luciferase activity was significantly inhibited in MEF2C-AS1-wt group (*p* < 0.01), but there was no change in MEF2C-AS1-mut group. Besides, qRT-PCR results elucidated that overexpression of lncRNA MEF2C-AS1 sharply reduced the expression of miR-592 (*p* < 0.01), confirming that miR-592 bound with lncRNA MEF2C-AS1 (Figure 3G).

### Overexpression of lncRNA MEF2C-AS1 Inhibited Proliferation, Migration and Invasion of CC Cells by Regulating miR-592

**FIGURE 4:**
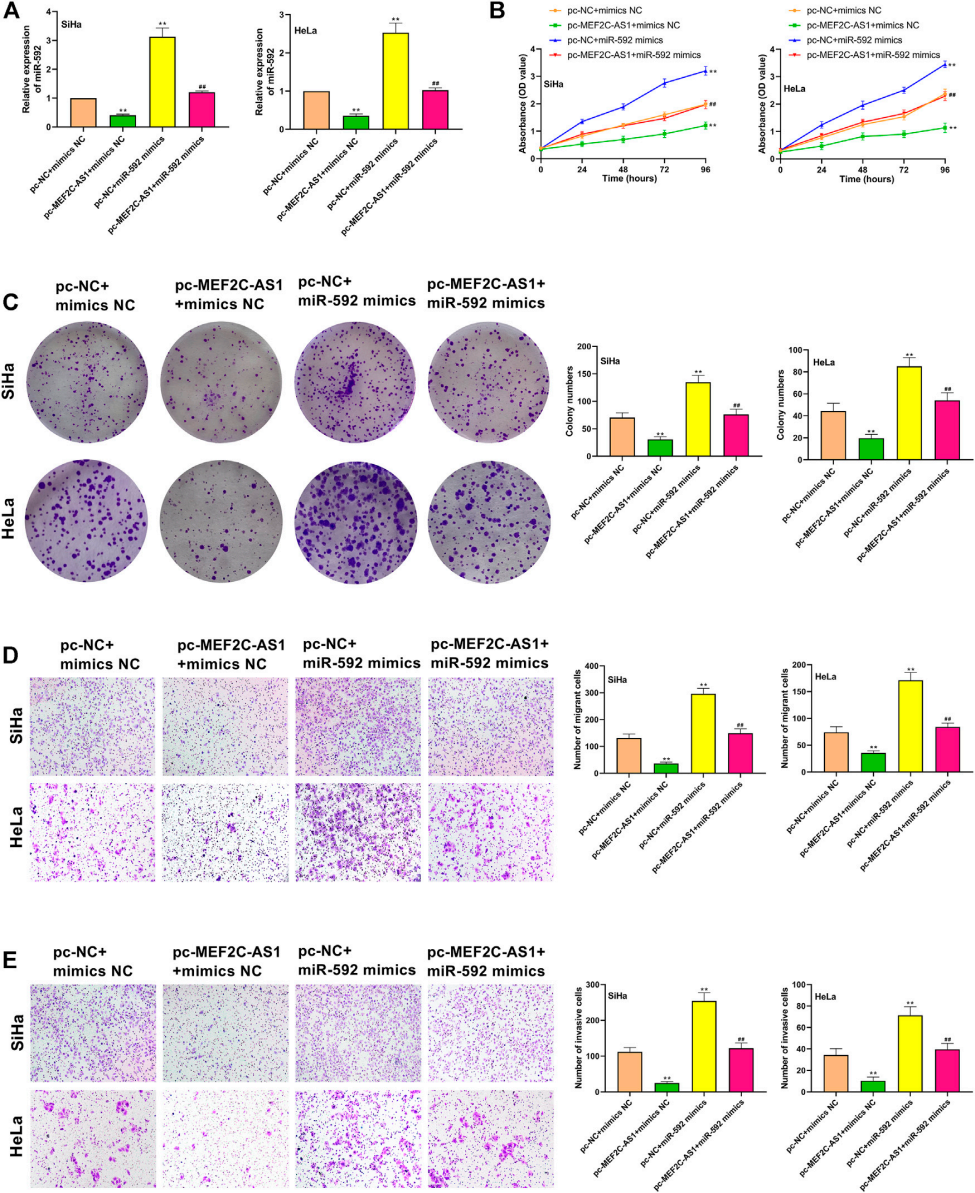
Overexpression of lncRNA MEF2C-AS1 inhibited proliferation, migration and invasion of CC cells by regulating miR-592. **(A)** Expression of miR-592 after transfection of pc-MEF2C-AS1, miR-592 mimics or pc-MEF2C-AS1 + miR-592 mimics was detected by qRT-PCR; **(B)** The proliferation ability of SiHa and HeLa after transfected with pc-MEF2C-AS1, miR-592 mimics or pc-MEF2C-AS1 + miR-592 mimics was measured by CCK-8; **(C)** The colony formation ability of SiHa and HeLa after transfected with pc-MEF2C-AS1, miR-592 mimics or pc-MEF2C-AS1 + miR-592 mimics was measured by colony formation assay; **(D)** After transfected with pc-MEF2C-AS1, miR-592 mimics or pc-MEF2C-AS1 + miR-592 mimics, transwell assay was conducted to detect the migration ability of SiHa and HeLa; **(E)** After transfected with pc-MEF2C-AS1, miR-592 mimics or pc-MEF2C-AS1 + miR-592 mimics, transwell assay was performed to measure the invasion of SiHa and HeLa. Measurement data were expressed as mean ± standard deviation. The cell experiment is repeated three times independently. **p < 0.01, compared with pc-NC + mimics NC group; ^##^ *p* < 0.01, compared with pc-MEF2C-AS1 + mimics NC or pc-NC + miR-592 mimics group **(A–E)**.

We had proved that lncRNA MEF2C-AS1 was the direct target of miR-592 in CC cells. To further demonstrate the role of the interaction between the above in CC, we transfected SiHa and HeLa cells with pc-MEF2C-AS1 or miR-592 mimics or pc-MEF2C-AS1 + miR-592 mimics. In Figure 4A, when compared with pc-NC + mimics NC group or pc-MEF2C-AS1 + miR-592 mimics group, the expression of miR-592 was significantly reduced in pc-MEF2C-AS1 + mimics NC group (*p* < 0.01) and remarkedly elevated in pc-NC + miR-592 mimics group (*p* < 0.01), revealing that the transfection was successful. Further, we examined the effects of transfection of these plasmids on cell behavior. CCK-8 and colony formation data showed that cell viability and cell clone numbers were significantly decreased after transfection with pc-MEF2C-AS1 (*p* < 0.01) (Figures 4B,C). However, when co-transfected with miR-592 mimics, its growth activity and the number of visible proliferating cells increased remarkably (*p* < 0.01) (Figures 4B,C). Similarly, in the migration and invasion tests, overexpression of lncRNA MEF2C-AS1 inhibited cell migration and invasion (*p* < 0.01) (Figures 4D,E). Conversely, after co-transfection with miR-592 mimics, the inhibition of lncRNA MEF2C-AS1 to miR-592 was partially offset (*p* < 0.01) (Figures 4D,E).

### RSPO1 Was the Target of miR-592

**FIGURE 5:**
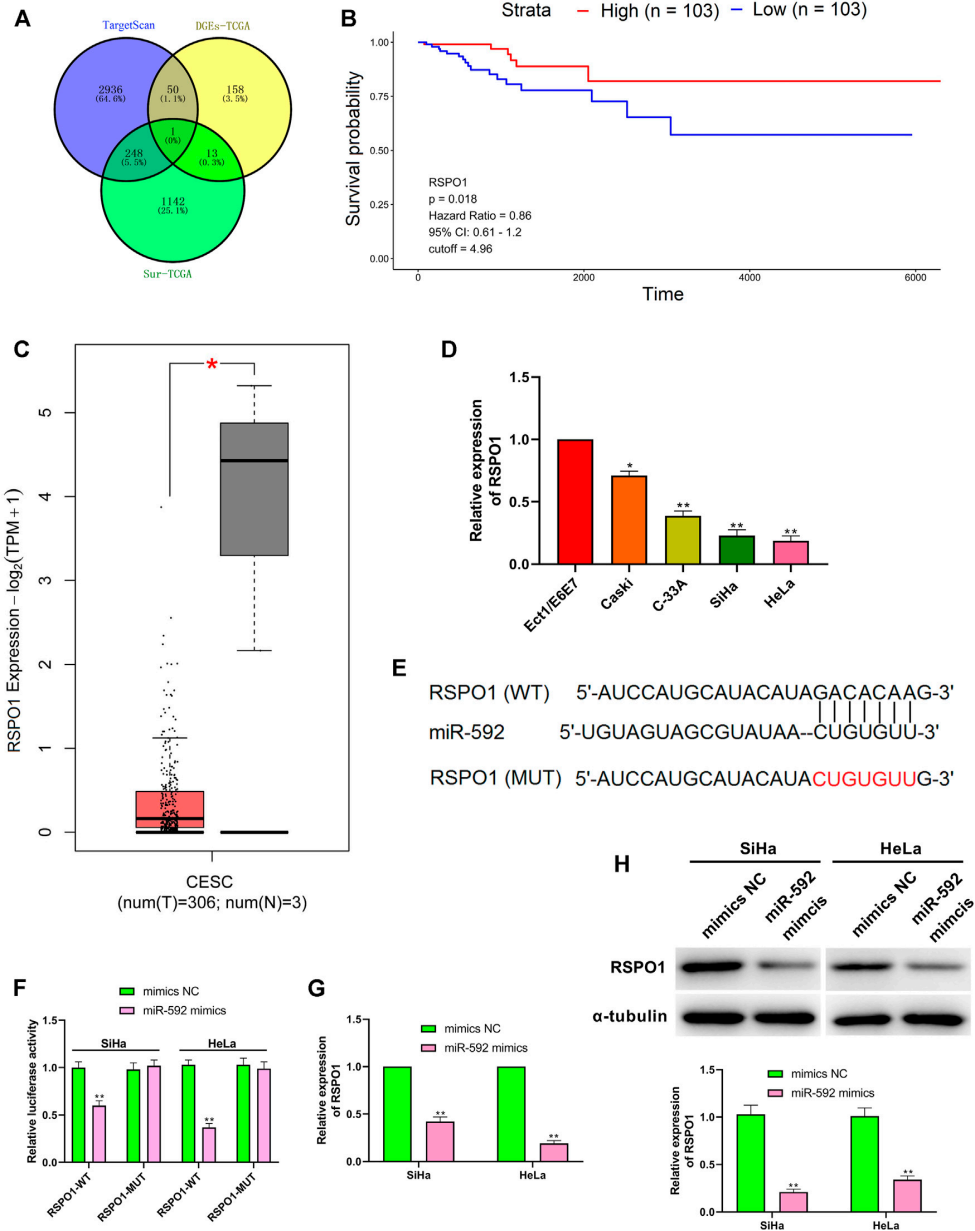
RSPO1 was the target of miR-592. **(A)** TargetScan, the differentially expressed mRNA screened from TCGA database and the mRNA with significant survival differences screened from TCGA database are used to predict RSPO1 can bind to miR-592; **(B)** Kaplan-Meier survival curve from TCGA described the overall survival (the criteria of two groups was Quartile); **(C)** The expression of RSPO1 in CC tissues compared with the normal tissues in the GEPIA website; **(D)** qRT-PCR was performed to detect the RSPO1 expression in cervical cancer cell lines (Caski, C-33A, SiHa, and HeLa) and normal cervical cell (Ect1/E6E7); **(E)** TargetScan predicts the binding sites between miR-592 and RSPO1; **(F)** luciferase activity of RSPO1-wt, RSPO1-mut upon the transfection of miR-592 mimics or mimics NC was evaluated by luciferase reporter assay; **(G)** The mRNA expression of RSPO1 was detected in SiHa and HeLa cells by qRT-PCR after transfection with miR-592 mimics; **(H)** The protein expression of RSPO1 was detected in SiHa and HeLa cells by qRT-PCR after transfection with miR-592 mimics. Measurement data were expressed as mean ± standard deviation. The cell experiment is repeated three times independently. *p < 0.05, compared with normal tissues **(C)**; *p < 0.05, **p < 0.01, compared with Ect1/E6E7 group **(D)**; **p < 0.01, compared with mimics NC group **(F–H)**.

As shown in Figure 5A, the target genes of miR-592 were predicted by retrieving the TargetScan, the differentially expressed mRNA of CESC screened from TCGA database and the mRNA with significant survival differences screened from TCGA database. Then, the genes were intersected using Venn analysis, and revealed that RSPO1 was the only target gene (Figure 5A). The TCGA database showed that high expression of RSPO1 in CESC patients was associated with higher survival (Figure 5B). Besides, the data from GEPIA website showed that the expression level of RSPO1 was significantly decreased in tumor tissues, compared with normal tissue (Figure 5C). Similarly, RSPO1 was down-regulated in CC cells (*p* < 0.01) (Figure 5D). Further, TargetScan predicted the downstream target of miR-592 and the results showed that RSPO1 was its direct target (Figure 5E). To verify this prediction, we carried out luciferase reporter assay and observed that the relative luciferase activity was significantly reduced after transfection with miR-592 mimics (*p* < 0.01) (Figure 5F). However, when this binding site was mutated, over-expression of miR-592 did not affect luciferase activity (Figure 5F). Besides, both qRT-PCR and western blot results illustrated that over-expression of miR-592 evidently inhibited RSPO1 expression at both transcriptional and translational levels (*p* < 0.01) (Figures 5G,H).

### Low Expression of miR-592 Inhibited Proliferation, Migration and Invasion of CC Cells by Regulating RSPO1

**FIGURE 6:**
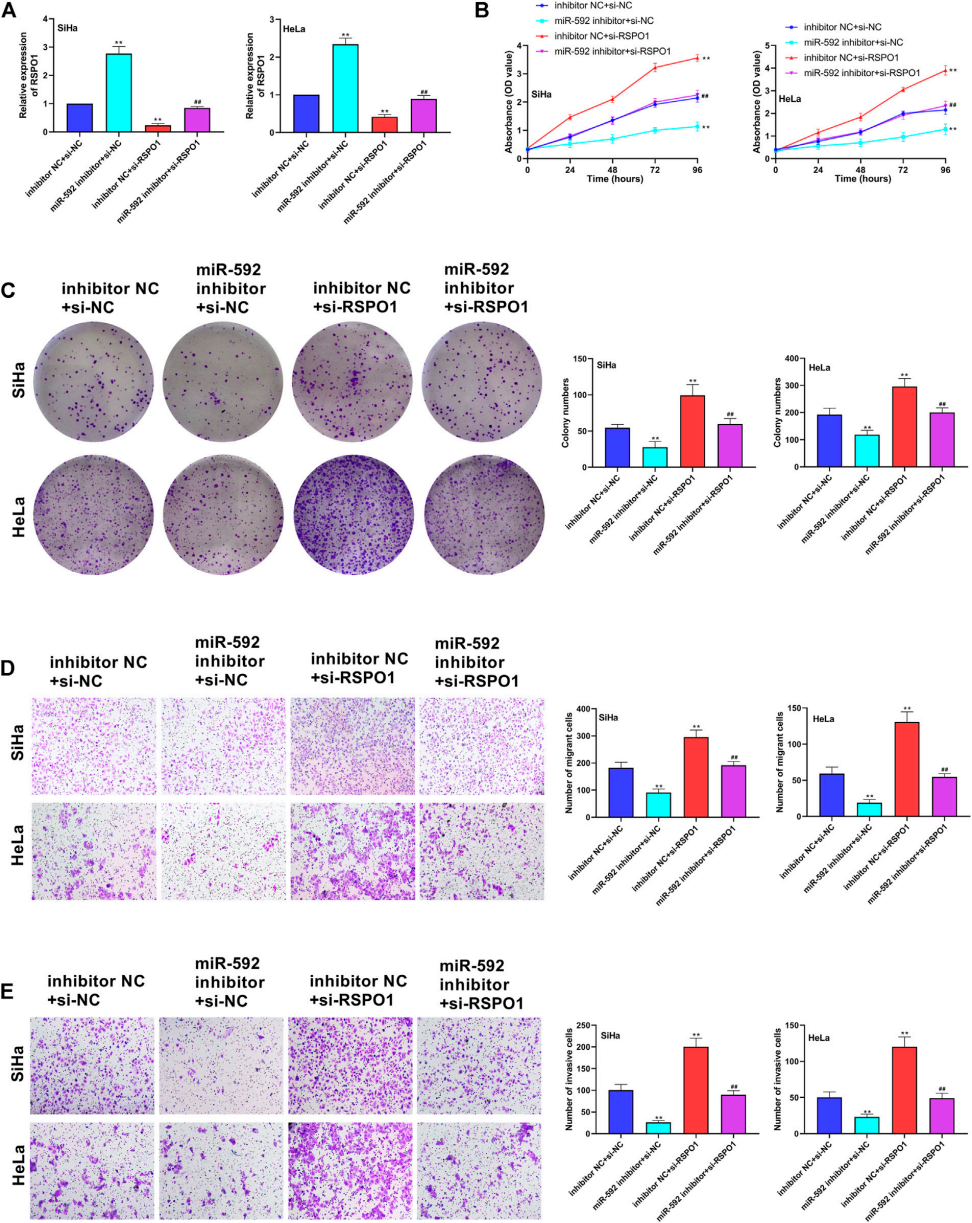
Low expression of miR-592 inhibited proliferation, migration and invasion of CC cells by regulating RSPO1. **(A)** Expression of miR-592 after transfection of miR-592 inhibitor, si-RSPO1 or miR-592 inhibitor + si-RSPO1 was detected by qRT-PCR; **(B)** The proliferation ability of SiHa and HeLa after transfected with miR-592 inhibitor, si-RSPO1 or miR-592 inhibitor + si-RSPO1 was detected by CCK-8; **(C)** The colony formation ability of SiHa and HeLa after transfected with miR-592 inhibitor, si-RSPO1 or miR-592 inhibitor + si-RSPO1 was measured by colony formation assay; **(D)** The migration ability of SiHa and HeLa after transfected with miR-592 inhibitor, si-RSPO1 or miR-592 inhibitor + si-RSPO1 was detected by using transwell assay; **(E)** The invasion ability of SiHa and HeLa after transfected with miR-592 inhibitor, si-RSPO1 or miR-592 inhibitor + si-RSPO1 was detected by using transwell assay. Measurement data were expressed as mean ± standard deviation. The cell experiment is repeated three times independently. **p < 0.01, compared with inhibitor NC + si-NC group; ^##^ *p* < 0.01, compared with miR-592 inhibitor + si-NC or inhibitor NC + si-RSPO1 group **(A–E)**.

To further explore the relationship between miR-592 and RSPO1 in the progression of CC, we transfected SiHa and HeLa cells with miR-592 inhibitor or si-RSPO1 or miR-592 inhibitor + si-RSPO1. As Figure 6A showed, when compared with inhibitor NC + si-NC group or miR-592 inhibitor + si-RSPO1 group, the expression of RSPO1 was significantly increased in miR-592 mimics + si-NC group (*p* < 0.01) and remarkedly decreased in inhibitor NC + si-RSPO1 group (*p* < 0.01), confirming that the transfection was successful. Subsequently, we examined the effects of transfection of these plasmids on cell behavior. CCK-8 and colony formation assay results depicted that after single transfection with miR-592 inhibitor, the proliferation activity of SiHa and HeLa cells was significantly enhanced (*p* < 0.01), while co-transfection with si-RSPO1, its proliferative activity decreased instead (*p* < 0.01) (Figures 6B,C). Similarly, in Figures 6D,E, we clearly saw that miR-592 inhibitor significantly inhibited the migration and invasion of cervical cancer cells (*p* < 0.01). After co-transfection with si-RSPO1, the inhibition of migration and invasion was offset to some extent (*p* < 0.01) (Figures 6B,C).

## Discussion

A mountain body of evidences has proved that the abnormal expression of lncRNAs is often associated with various types of cancer, including CC (Fatica and Bozzoni, 2014; Xie et al., 2019). As shown in the study of Gao et al., lncRNA HAND2-AS1 inhibited CC progression via targeting miRNA-21-5p/TIMP3/VEGFA (Gao et al., 2020). Beside, LINC00636 promoted lymph node metastasis and CC through regulating NM23 (Zhong et al., 2020). In the presented study, we observed that lncRNA MEF2C-AS1 was up-regulated in CC tissues and cell lines, and the high expression was also associated with the high survival rate. Early study reported that lncRNA MEF2C-AS1 inhibited the proliferation, migration and invasion of breast cancer cells by reducing the expression of miR-3646 (Bing et al., 2019). In diffuse gastric cancer, lncRNA MEF2C-AS1 was regard as a novel biomarker (Luo et al., 2018). However, there is almost no research on lncRNA MEF2C-AS1 in CC. To further investigate the biological role of lncRNA MEF2C-AS1 in CC, we conducted functional studies. The results depicted that the overexpression of lncRNA MEF2C-AS1 inhibited proliferation, migration, and invasion of CC cells. Taken together, lncRNA MEF2C-AS1 is involved in the progress of CC.

Functionally, the lncRNA molecule is rich in microRNA (miRNA) binding sites, which acts as a miRNA sponge in cells, thus removing the inhibition of miRNA on its target genes and increasing the expression level of target genes (Adams et al., 2017). Through interacting with miRNAs associated with disease, lncRNAs play an important regulatory role in disease. Previously, miR-592 has been reported to promote gastric cancer progression through PI3K/AKT and MAPK/ERK signaling pathways (He et al., 2018). In breast cancer, miR-592 has also been reported to inhibit breast cancer progression by inhibiting TGF-β2 (Hou et al., 2017). In this study, we used TGCA database and DIANA to predict that lncRNA MEF2C-AS1 was the target of miR-592 and the direct binding between them was confirmed by double fluorescence assay. To further verify the biological relationship between lncRNA MEF2C-AS1 and miR-592 in CC, we then carried out functional studies. As expected, after transfection with pc-MEF2C-AS1, the expression of miR-592 was significantly down-regulated and the proliferation, migration and invasion ability of cancer cells were significantly reduced. On the contrary, the expression of miR-592 gradually increased after co-transfection with miR-592 mimics. Accordingly, the proliferation activity, migration and invasion of cancer cells are enhanced. Taken together, these above results indicated that lncRNA MEF2C-AS1 acted as a sponge of miR-592 to regulate CC.

R-spondin1 (RSPO1) is a 29kd, 263 amino acid protein. Initial studies have shown that RSPO1 is expressed by intestinal endocrine cells in various tissues. It is a potent and specific epithelial mitogen that can stimulate the growth of the small and large intestine mucosa (Kim et al., 2005; Zhao et al., 2007). But recent data shows that RSPO1 is also expressed in other tissues and certain cancer cells. Zhao et al. showed that RSpo1 protein therapy increased the thickness of oral mucosa after irradiation and chemotherapy, reduced ulcers, and protected mice from oral mucositis caused by chemotherapy or radiation (Gregorieff and Clevers, 2005). Besides, in the mammary gland, Rspo1 and Wnt4 cooperate to promote the self-renewal of basal breast stem cells (Cai et al., 2014). In our study, we found that RSPO1 was significantly reduced in CC tissues and cells, and was negatively correlated with miR-592 expression. Further, rescue experiment verified that low expression of miR-592 inhibited proliferation, migration and invasion of cervical cancer cells by regulating RSPO1. Taken together, our results indicated that the role of lncRNA MEF2C-AS1 in CC may be related to the expression of miR-592/RSPO1.

## Conclusion

In this study, we mainly explored the functionality of lncRNA MEF2C-AS1 and its potential interactions with miR-592/RSPO1 axis in CC. We found that lncRNA MEF2C-AS1 was down-regulated in CC and may regulate miR-592/RSPO1 axis to participate in the regulation of CC cell invasion and migration.

## Data Availability

The raw data supporting the conclusion of this article will be made available by the authors, without undue reservation.
